# An anatomical-like triangular-vector ligament reconstruction for the medial collateral ligament and the posterior oblique ligament injury with single femoral tunnel: a retrospective study

**DOI:** 10.1186/s13018-017-0602-3

**Published:** 2017-06-26

**Authors:** Hongtao Xu, Kai Kang, Jian Zhang, Dongmei Xin, Wei Liu, Guorong Jin, Jiangtao Dong, Shijun Gao

**Affiliations:** 1 Department of Joint Surgery, The Third Hospital of Hebei Medical University, NO. 139 Ziqiang Road, Shijiazhuang, 050051 Hebei People’s Republic of China; 2 People’s Hospital of Ri Zhao, Taian Road, Rizhao, 276800 Shandong People’s Republic of China; 3 Hospital of TCM, 35 Wanghai Road, Rizhao, 276800 Shandong People’s Republic of China

**Keywords:** Medial collateral ligament, Posterior oblique ligament, Anatomical reconstruction, External rotation stability

## Abstract

**Background:**

The purpose of this study was to evaluate the clinical outcomes of anatomical-like triangular-vector ligament reconstruction (TLR) in treating the combined injury of medial collateral ligament (MCL) and posterior oblique ligament (POL).

**Methods:**

During July 2013 to May 2014, 26 patients who received anatomical-like TLR were included into this study. All patients received clinical physical examination, imaging examination, and knee joint function score both preoperative and follow-up. The stability of the medial structure of the knee joint was examined by physical examination and imaging evaluation, including excessive knee medial opening (EKMO) and tibial external rotation angle (TERA). The function of the knee was evaluated by the subjective questionnaire, including Lysholm, Tegner, and IKDC score. SPSS software was used for statistics analysis.

**Results:**

The mean follow-up time exceeds 24 months. Two patients occurred with serious heterotopic ossification, and one patient received revision because of screw breakage. EKMO over the contralateral state at 0° decreased from 9.76 ± 2.76 mm to 2.79 ± 1.02 mm with statistical significance (*P* < .001) and 10.32 ± 2.75 mm decreased to 3.13 ± 0.85 mm at 30° (*P* < .001). Meanwhile, TERA significantly decreased from 53.38 ± 6.71° to 27.15 ± 4.92° (*P* < .001). The postoperative Lysholm, Tegner, and IKDC score were superior to preoperative with statistical significance (*P* < .001).

**Conclusions:**

Anatomical-like TLR can reconstruct the graft to cover the insertions which can regain anatomic form and function with a cramped space. Not only the valgus stability and rotational stability can be restored obviously at follow-up but also the usage of implantation can be reduced, decreasing the incidence rate of allergy and saving costs.

## Statement of clinical significance

This study provides evidence of the superiority of anatomical-like triangular-vector ligament reconstruction (TLR) in treating the combined injury of medial collateral ligament and posterior oblique ligament.

## Background

The medial collateral ligament (MCL) is one of the most commonly injured ligamentous structures [1]. It serves as the primary medial static stabilizer against valgus stress, though along with the posteromedial corner provides resistance to external rotation forces applied to the lower extremity [2]. Those sports involve valgus knee loading, such as hockey, skiing, and football, have contributed to the frequent occurrence of MCL injuries [3]. Both MCL and posterior oblique ligament (POL) are two main static stabilizers [4]. And the combined injury could result in clinically significant valgus or rotational instability [5]. Michael et al. reported that Hughston’s grade III MCL injury often result with a high risk up to 78% of concomitant ligament injury [6]. Of these cases, 95% involve with the ACL, may lead to chronic instability followed by disability [7, 8]. A better recovery of valgus and rotational stability was essential for massive MCL injury, let alone one who suffer with grade III MCL injury combine ACL injury.

Previous anatomical studies have demonstrated the relative position was unparalleled on different planes between MCL and POL [9, 10]. When the knee extended, the MCL runs parallel to the axis of the femur/tibia and the POL formed an angle of 25° with the axis of the femur/tibia. Since non-parallel, the extension lines of these two ligaments would intersect at one point at superior of femoral condyle.

This study shows the surgical procedure of an anatomical-like triangular-vector ligament reconstruction (TLR) technical of the MCL and POL which bring a satisfied result of medial knee stability.

## Methods

### Participants

The retrospective study (level of evidence 3) was conducted with the approval of the ethics committee of The Third Hospital of Hebei Medical University, Shijiazhuang, China. From July 2013 to May 2014, 47 patients suffered with unilateral MCL injury in our institute. The inclusion criteria were (1) simple injury of the MCL, (2) preoperative magnetic resonance imaging (MRI) that confirmed the MCL rupture, and radiographic stress position imaging showed the excessive knee medial opening over the contralateral state (EKMO) was more than 3 mm compared with contralateral knee [11, 12], (3) the valgus stress test was positive at 0° and 30° knee flexion, (4) no previous knee surgery, and (5) with whole clinical follow-up data. The surgical indications for anatomical-like TLR were (1) chronic MCL injury; (2) Hughston grade III or above sub-acute or acute MCL injury with posterior-medial structure injury (the valgus stress tests showed both positive at 0° and 30° knee flexion) [12–14]. Six (12.8%) of them were excluded from the study because they combined with ACL injury which have an effect on the rotational instability. In total of 16 patients who suffered acute MCL injury were not suited in this study. However, five (19.2%) included patients whose injury to operation interval were fewer than 3 days because their EKMO over the contralateral state were more than 10 mm both at 0° and 30° knee flexion. This massive valgus laxity was considered as high grade MCL injury which needs to be treated with surgical repair or reconstruction. In addition, 17 (65.4%) chronic patients and 4 (15.4%) sub-acute patients were included into this study. So there were total 26 patients received anatomical-like TLR and be included into this study. General patient information is listed in Table 1.

**Table 1:** Demographic characteristics and intraoperative data

Basic information		Data
Age, mean ± SD, years		27.42 ± 4.19
Sex, male:female, *n*		21/5
Side, left/right, *n*		11/15
BMI, mean ± SD, kg/m^2^		27.47 ± 6.20
Injury to operation interval, mean ± SD, *days*		35.88 ± 20.26
EKMO over the contralateral state, mean ± SD, millimeters	0°	9.76 ± 2.27
	30°	10.32 ± 2.76
TERA, Mean ± SD, degrees		53.38 ± 6.71
Follow-up, mean ± SD, months		24.38 ± 3.23

*BMI* body mass index, *EKMO* excessive knee medial opening, *TERA* tibial external rotation angle, *n* number

### Study procedures

**Fig. 1:**
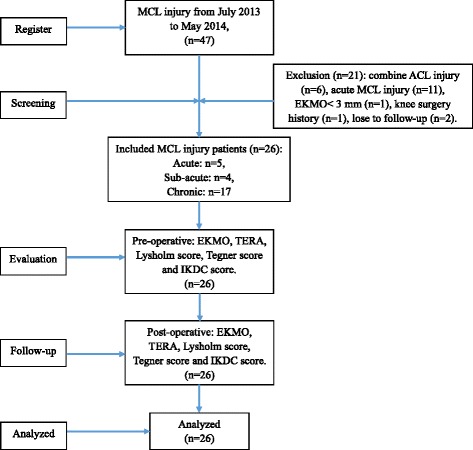
Consolidated Standards of Reporting Trials (CONSORT) flowchart

The data were collected from the resident’s admission note, physical examination, preoperative radiographic stress position imaging, operation records, and records of pre- and post-operative functional scores. Patients were evaluated using the Lysholm score, Tegner activity level, and International Knee Documentation Committee (IKDC) Knee Evaluation Form before the operation (Fig. 1). An average of 24.4-month follow-up, 26 patients returned to complete the same examination and evaluation as performed preoperatively.

### Surgical technique

#### Physical examination and arthroscopic evaluation

**Fig. 2:**
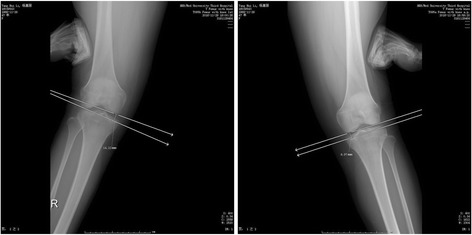
Bilateral EMKOs were applied and measured at different angle of knee flexion. This figure shows the EMKO at 0°

**Fig. 3:**
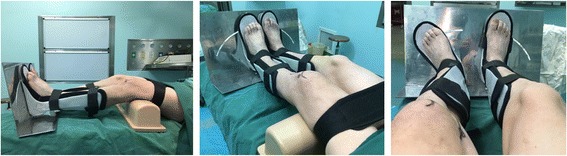
The knee rotation angle was measured by the TERA

After anesthesia, the medial structures were evaluated by a surgeon. To detect medial joint opening, EMKO was applied at 0° and 30° of knee flexion (Fig. 2). The estimation of knee rotation was assessed in comparison with the contralateral knee which profited from the author’s patent called tibial external rotation angle (TERA) Measuring Instrument (201620091253.9) (Fig. 3). Whether other structure were injured or not, they should be eliminated and dealt with during arthroscopic evaluation. FasT-Fix was considered as the first choice to deal with meniscal lesion. If the meniscal tear type was difficult to suture, meniscectomy was performed.

#### Preparation of allograft

A thawed allograft should be soaked for 20 min. The graft was measured to make sure no less than 24 cm in length and 5 mm in diameter, meanwhile, the diameter of the combined ends was no less than 7 mm. Because of the relevance between the graft length and tunnel depth, only one free end was braided with no. 2 Ethibond Excel Polyethylene non-absorbable sutures. Then a thread was passed through combined graft and was looped around which can guide the sutured end.

#### Reconstruction procedure of MCL–POL

##### Locating the insertions of MCL and POL

**Fig. 4:**
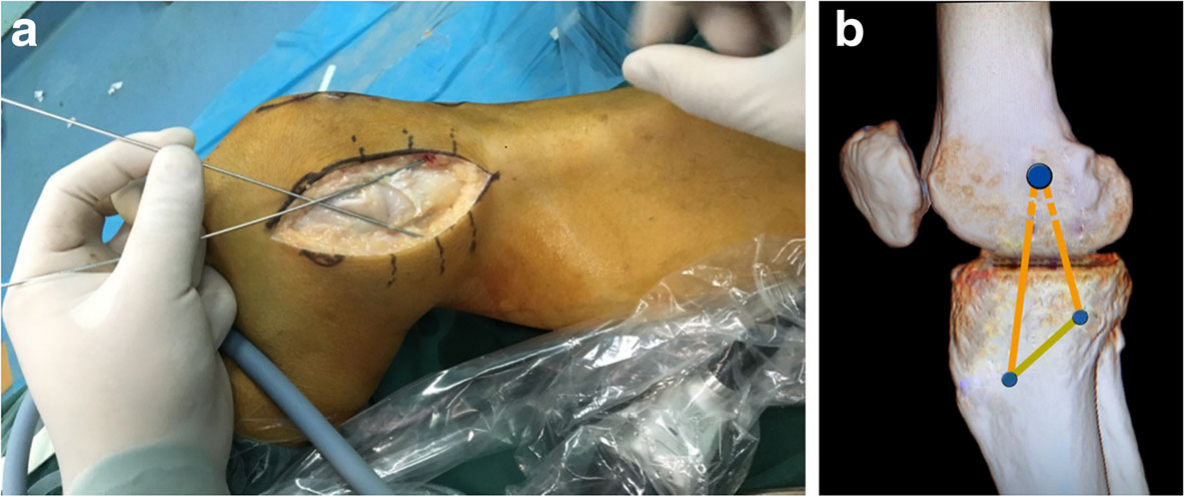
**a** Two Kirschner wires (K-wires) were used to locate and link the MCL and the POL insertions of tibia and femur, respectively. **b** They had an intersection point which was the drilling location of the femoral tunnel

A curved medial skin incision was directed from 1 cm above the adductor tubercle down to 6 cm beyond the joint line. Generally observing on the MCL and POL, then locating insertions respectively. The tibial MCL insertion was selected at the place 1 cm anterior the narrowing point of posterior tibial ridge and 4.5 cm below the tibia plateau. The tibial POL insertion is selected at 2 mm lateral of the medial tibia and 2 cm below the tibia plateau. After, the medial femoral epicondyle was exposed and both the femoral MCL and the POL insertion sites were identified. The femoral MCL insertion was approximately selected at the anterior inferior part of adductor tubercle of condyle of femur, 3 mm below the proximal medial epicondyle of femur and 5 mm anterior the posterior edge of medial epicondyle of femur. The femoral POL insertion was selected at 8 mm below the medial epicondyle of femur and 6 mm anterior the posterior edge of medial epicondyle of femur that is closed to the femoral anatomical attachment points of the MCL. Two Kirschner wires (K-wires) were used to locate and link the MCL and the POL insertions of tibia and femur, respectively. The two K-wires had an intersection point which was the drilling location of the femoral tunnel (Fig. 4).

##### Drilling tibial and femoral tunnel

**Fig. 5:**
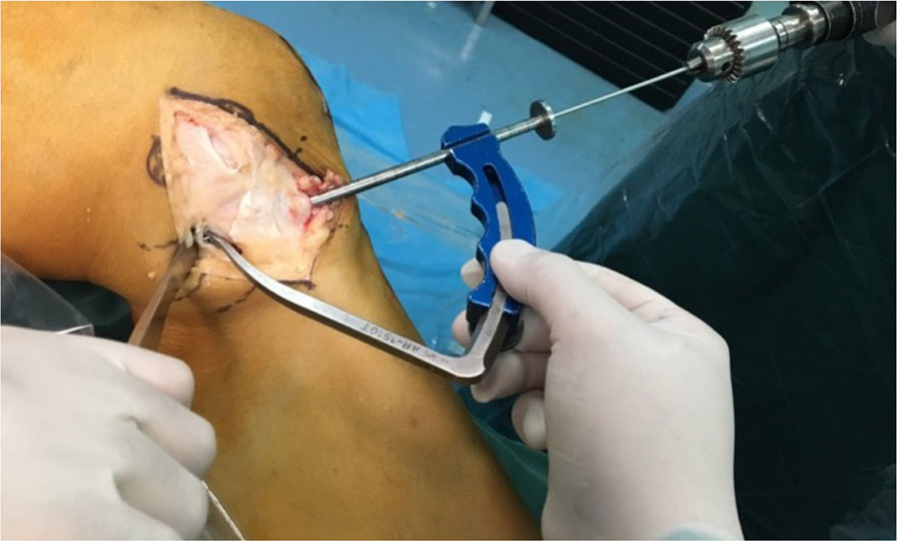
A guide apparatus was used for drilling tunnel accurately

**Fig. 6:**
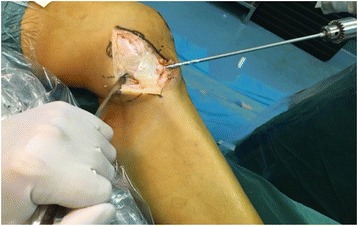
The tibial tunnel was broadened by a bone pin of 5 mm in diameter

The tibial tunnel linked the center situs of two ligament insertions. A 2-mm guide pin was oriented by a guide apparatus which was used for cruciate ligament reconstruction (Fig. 5). And then the tibial tunnel was broadened by a 5-mm bone pin (Fig. 6). The sutured graft end was pulled through the tunnel by the previous guide pin.

**Fig. 7:**
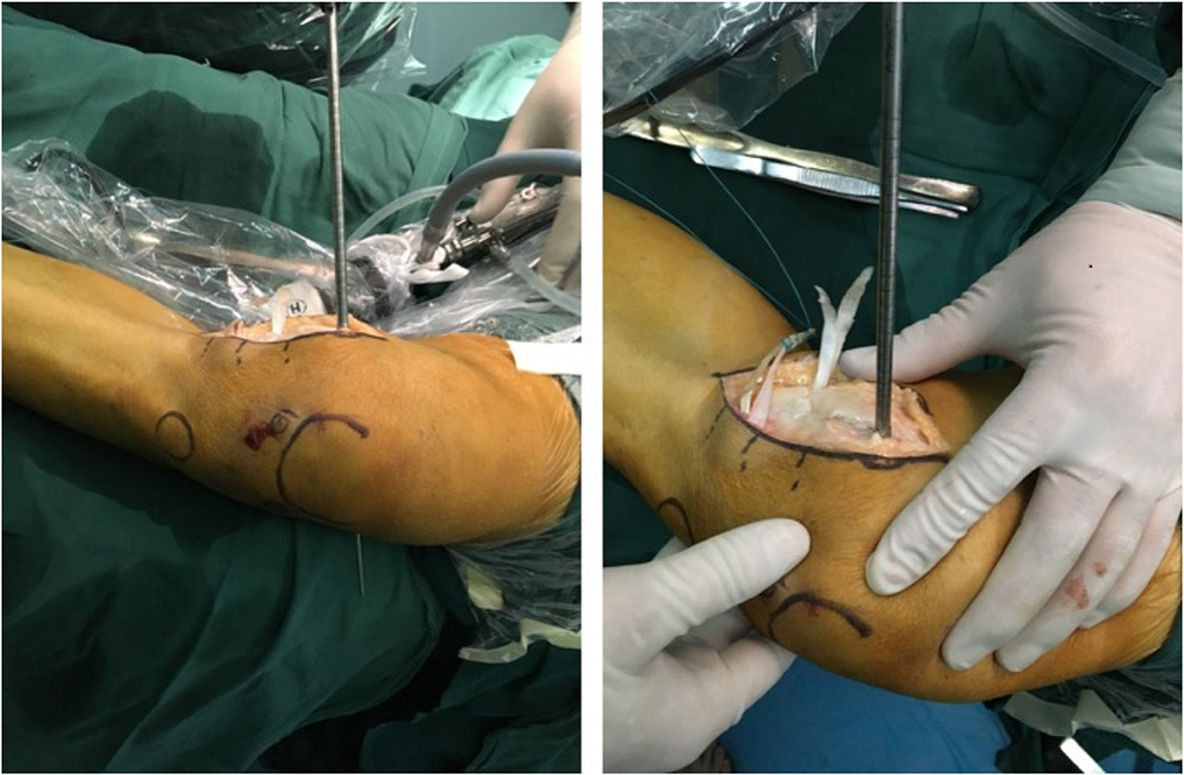
A thick bone pin was drilled approximately 2.5 cm in depth

**Fig. 8:**
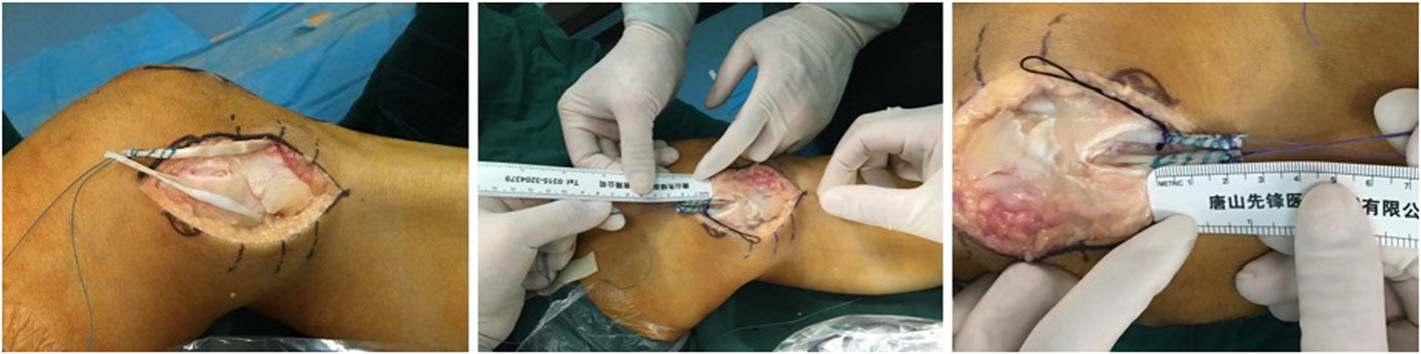
The graft was measured again and then sutured the non-sutured end

The femoral drilling site had been located by the preceding K-wires’ intersection point. Another guide pin was drilled into the intersection point along the epicondylar axis, which formed an angle of 30° with sagittal plane and came out at the lateral condyle of the femur. So the intercondylar notch could avoid from being crossed by the pin. A 7-mm bone pin was drilled approximately 2.5 cm in depth along with the previous guide pin which could accommodate the femoral attachments of the graft (Fig. 7). The graft was measured a second time after running through the tunnels. Then the non-sutured end could be sutured with an appropriate length (Fig. 8).

##### Graft passage and fixation

**Fig. 9:**
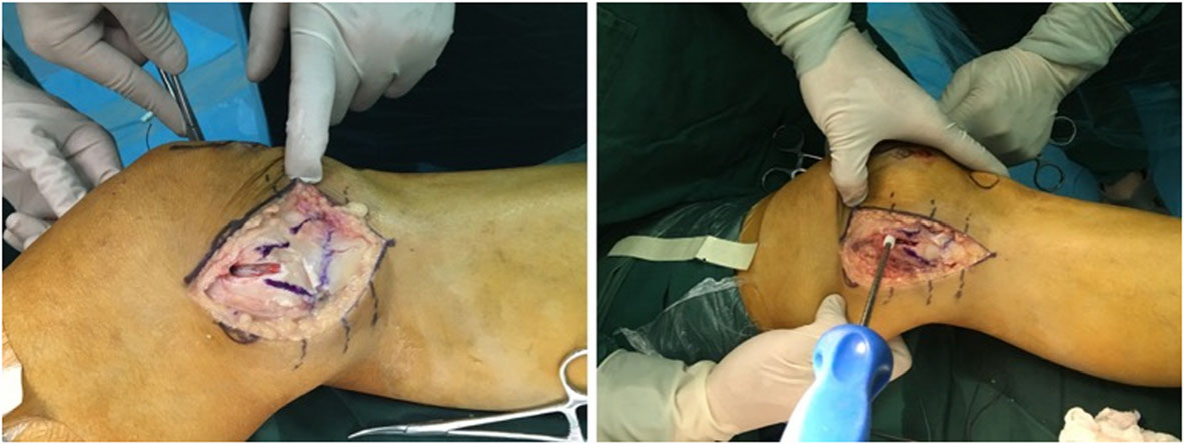
A bio-interference screw was screwed into the tunnel entrance

The two free ends of the graft were pulled through the femoral tunnel, respectively, by guide pin. A tensile force was provided when the knee was kept at 30° flexion with varus stress and neutral rotation. A bio-interference screw with the same size as the femoral tunnel was screwed into the tunnel entrance where the graft was choked (Fig. 9). The graft was sutured to the surrounding soft tissue at the two tunnel exits in tibial which could prevent sliding and impact between the graft and bone tunnel.

##### Arthroscopic reexamination and stitching up

Arthroscopic evaluation was performed again to confirm the intrinsic femur-tibia gap was no more expanded. Then the wound was thoroughly irrigated and sutured.

### Postoperative treatment and rehabilitation

Patients were injected with cefazolin sodium pentahydrate every 6 h during the first 24 h. After the anesthetic effect dissipated, the patient needed to practice ankle pump as earlier as possible. Not only the swelling could be reduced but also the incidence rate of deep venous thrombosis could be declined.

The patient received a long hinged brace with no weight bearing for 6 weeks. Non-weight-bearing walking was encouraged, but the long hinged brace should keep equipped. During this period, range of motion (ROM) exercises were restricted from 0° to 90° of knee flexion. Six weeks later, knee flexion progressed to a full ROM and weight-bearing walking was allowed as tolerated and mobilization without brace protection was permitted. Patients could do further controlled activities after 3 months and contact sports after 6 months.

### Follow-up

At follow-up, 26 patients were re-examined clinically using the EKMO over the contralateral state, TERA, Lysholm Score, Tegner Activity Level, and IKDC. The mean follow-up period was 24.38 ± 3.23 months.

### Statistical analysis

Data were analyzed using SPSS software for Windows (version 21.0; Chicago, IL). Wilcoxon matched-pairs signed-rank test (non-parametric) was used to compare the difference in the positive rate for the preoperative and follow-up data. The significance level was set at *P* < .05.

## Results

A total of 26 patients (21 males and 5 females) with a mean age of 27.42 ± 4.19 years were analyzed in this study. Patient demographic data are listed in Table 1. There were 24 cases whose EKMO over the contralateral state widened up to 5 mm at 0°, and all 26 cases’ EKMO over the contralateral state were widened up to 5 mm at 30°.

At a mean 24.4-month follow-up time, significant differences were observed between the preoperative and postoperative data for all of these measures (*P* < .001). EKMO over the contralateral state at 0° decreased from 9.76 ± 2.76 mm to 2.79 ± 1.02 mm with statistical significance (*P* < .001) and 10.32 ± 2.75 mm decreased to 3.13 ± 0.85 mm at 30° (*P* < .001). Meanwhile, TERA significantly decreased from 53.38 ± 6.71° to 27.15 ± 4.92° (*P* < .001). The subjective evaluation and activity level scores, included Lysholm, Tegner, and IKDC score, increased with statistical significance (*P* < .001). The data are listed in Table 2.

**Table 2:** EKMO over the contralateral state, TERA, subjective evaluation, and activity level scores before and after surgery

		Preoperative mean ± SD (95%CI)	Follow-up mean ± SD (95%CI)	*Z*	*P*
EKMO over the contralateral state	0°	9.76 ± 2.27 (8.84-10.67)	2.79 ± 1.02 (2.38-3.20)	−4.457	<.001^a^
	30°	10.32 ± 2.76 (9.21-11.44)	3.13 ± 0.85 (2.78-3.47)	−4.458	<.001^a^
TERA		53.38 ± 6.71 (50.68-56.09)	27.15 ± 4.92 (25.17-29.14)	−4.460	<.001^a^
Lysholm		49.42 ± 5.32 (47.28-51.57)	90.35 ± 4.55 (88.51-92.18)	−4.461	<.001^a^
Tegner		1.65 ± 0.56 (1.43-1.88)	5.77 ± 0.86 (5.42-6.12)	−4.498	<.001^a^
IKDC		47.85 ± 5.17 (45.76-49.93)	87.88 ± 3.62 (86.42-89.34)	−4.463	<.001^a^

*EKMO* excessive knee medial opening, *TERA* tibial external rotation angle, *IKDC* international knee documentation committee knee evaluation form, *CI* confidence interval

A Wilcoxon matched-pairs signed rank test (non-parametric) was used to compare the difference in the positive rate for the preoperative and follow-up data

^a^Statistically significant

### Complications

**Fig. 10:**
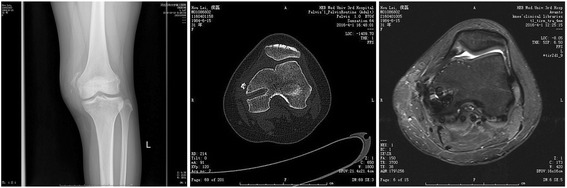
Post-operative heterotopic ossification at the femoral tunnel can be diagnosed by X-ray, CT scan and MRI scan, respectively

Two patients (7.7%) complained knee joint medial pain because heterotopic ossification occurred in the inlet of femoral tunnel (Fig. 10). Analgesic plaster was used for conservative treatment and no further complaint. One revision (3.8%) had failure of fixation in the femoral tunnel because of the screw breakage. There was no graft rejection and infection during follow-up.

## Discussion

Currently, the treatment for the MCL and POL injury is a broad academic controversy. However, a significant proportion of surgeons reached a consensus that Hughston’s grade III MCL injury, which were considered as a massive MCL and POL injury, need to be treated with surgical repair or reconstruction because there was a high risk of valgus and rotational instability [11, 12, 15]. This study elaborated the method of anatomical-like TLR of the MCL and POL and proved the better recovery of medial stability and clinical function.

Numerous surgical treatment procedures had been described with satisfactory clinical results [3, 13, 16–18, 19–22]. As the typical surgical methods for medial instability, Lind et al. [13], Yoshiya et al. [22], and Borden et al. [18] reported a double-bundle graft technique which can restore critical medical valgus stability. However, there were limited data available to evaluate their effectiveness of such procedures in improving rotational stability. We speculated that these treatments might ignore the high grade MCL injury that could combine posterior-medial structure damage, especially that the POL plays a secondary structure in resisting external rotational stability [4, 5]. After comparing the anatomic ligament repair (ALR) and TLR in treating grade III MCL injury in earlier stage works, Dong et al. found that TLR offered better rotatory stability than ALR at final follow-up [23]. In our study, the TERA significantly decreased from 53.38 ± 6.71° to 27.15 ± 4.92° (*P* < .001) which proved the effectiveness of restoring rotational stability. Both at 0° and 30° knee flexion, the EKMO over the contralateral state decreased with significant difference (*P* < .001 both at 0° and 30° knee flexion). It also demonstrated that the anatomical-like TLR can obtain satisfactory results in respect of restoring valgus stability.

Even though most included patients (17/26, 65.4%) in our study were chronic injury, still 5 (19.2%) patients with acute injury received anatomical-like TLR. A great majority of surgeon supported that non-surgical treatment of acute injury and surgical repair or reconstruction of those ineffective cases with chronic processing injury [14]. Because of the reliable self-healing ability, the injured MCL could attain satisfactory clinical results with proper rehabilitation [3, 16]. But high grade MCL injury was commonly held as a massive injury which often combined with POL or posterior-medial structure [5]. Because of the muscle atrophy, derangement, and scar healing, the high grade MCL injury could not gain a satisfactory clinical result as good as low grade MCL injury without concomitant structure injury [24]. So some authors advocated acute surgical repair or reconstruction for cases with grade III or above laxity [13, 16, 25]. Dong et al. put forward similar point that 3° MCL injury could hardly yield a satisfactory result if only with revision scarring and incomplete healing. Moreover, if MCL and ACL injuries are combined, the ACL and MCL surgical procedures could not be separated because the medial instability was harmful to the ACL tendon-bone healing and early stage rehabilitative exercises [14]. That is also a similar reason we adopted a more radical approach to the high grade MCL injury combined with posterior-medial structure injury. Because extensive posterior-medial structure injury leads to massive instability and isolated MCL self-healing was unsatisfactory.

Previous studies have confirmed that anatomical reconstructions better restore normal knee biomechanics than non-anatomical reconstructions [13]. Coobs et al. demonstrated that anatomical medial reconstruction of MCL-POL to their insertions resulted in nearly normal biomechanical knee stability and satisfying prognosis [26]. Many surgeons select drilling multi-tunnels into each of the insertions. Weimann et al. described a technique with two tibial tunnels which were located at the insertions of MCL and POL [27]. However, on the tibia two eyelet pins are drilled from medial to lateral. Even though the lateral tunnel export was set below the baseline which can reduce the damage rate of endangering the peroneal nerve, long tubular bone was not a nutritious place for tendon-bone healing. So the allograft fixation could not get a tendon-bone healing firm of long-term outcome after operation. Liu et al. reported a technique with two femoral tunnels which were also located at the insertions. But two anatomical femoral tunnels would be inappropriate [28]. The major reason was the cramped space cannot accommodate two anatomical tunnels with a 2-mm bone wall between them. At least 2 mm thickness of the bone wall between two tunnels was required to retain a safe reconstruction [29]. According to LaPrade et al. report, the location of the MCL and POL insertions, from the geometric point of view, the distance between the two insertions’ center on the femur was approximately 5 mm [9]. If the 2-mm bone wall was kept entirely, there was only 1.5 mm left on each side. Generally, the diameter of the suitable size of bio-interference screw was 7 mm. If the center of the tunnel was located into the center of these two insertions, the 3.5-mm screw radius would definitely devour the bone wall. And worse, multi-tunnel had a high potential destroying accessory structures. Compared with the 7 mm screw, the single bundle allograft only had 5 mm in diameter. The graft grated with high-risk while screwing in. Dong et al. reported the original technique of triangular-vector reconstruction, who essentially abandoned the conception of anatomical reconstruction, especially the femoral tunnel was not selected at the anatomical insertion [30]. The femoral tunnel was located at the rotatory center of the knee that had been considered as isometric point. The anatomical reconstruction of the MCL was not taken into account, let alone reconstructed the function of POL. What truly matters is when the guide pin drilled paralleled with the joint line along the epicondylar axis, the intercondylar notch and the femoral insertion of PCL can be particularly vulnerable.

The greatest benefit of our technical procedure was that the tunnel on the tibia can drill through the two centers of MCL and POL insertion without transfixion. The femoral intersection point was located as the drilling center of femoral tunnel. Not only the cramped space can be solved but also the graft would cover the insertions which can regain anatomical form and function. Thus it can be called an anatomical-like reconstruction. In our study, we found the subjective evaluation and activity level scores, including Lysholm, Tegner, and IKDC score, increased with statistical significance (*P* < .001). On the other side, it also proved that the anatomical-like TLR can gain good clinical function. Furthermore, reducing the usage of implantation can decrease the incidence rate of allergy and save costs.

Some studies have reported heterotopic ossification in the medial part of knee which could cause pain and tenderness [15, 31]. However, heterotopic ossification could not be distinguished whether it is caused by the MCL site injury or by the post-operative friction at bone-graft interface around the tunnels. Meanwhile, no study can explain the reason for pain or tenderness after a long post-operative period. Actually, the heterotopic ossification was probably due to medial chronic inflammation post-operatively. Dong et al. suggested the surgeon to suture the graft to the surrounding soft tissue in tibial to prevent sliding [14]. But in our study, we found that even though the screw had been fixed at the femoral tunnel, the friction and impact still occurred at the bone-graft interface which might lead to chronic inflammation as the knee moves from flexion to extension and causes heterotopic ossification. So according to our experience, we highly suggest that surgeons better suture both the femoral side and tibial side grafts with the surrounding soft tissue to diminish the friction and impact which might reduce the incidence rates of heterotopic ossification.

### Limitation

The limitation includes three points. The first is the intersection point of those two K-wires could be located relatively high in some cases. The femoral insertion of medial patellofemoral ligament (MPFL) might be interfered by the bone drill. This is due to the distance of two femoral insertions is approximately 10 mm [32].While measuring, the K-wire which linked the POL insertions could be appropriately slopped a bit more because of the three separate arms of POL. The second limitation is that long-term outcomes need a minimum follow-up of 3 years which can prove the effectiveness of the modified technique. Moreover, the third limitation is that the superiority of anatomical-like TLR and non-anatomical site TLR needs to be researched.

## Conclusions

Anatomical-like TLR can reconstruct the graft to cover the insertions which can regain anatomical form and function with a cramped space. Not only the valgus stability and rotational stability can be restored obviously at follow-up but also the usage of implantation can be reduced, decreasing the incidence rate of allergy and saving costs.

## Abbreviations

ALR: Anatomic ligament repair
EKMO: Excessive knee medial opening
IKDC: International Knee Documentation Committee
MCL: Medial collateral ligament
MPFL: Medial patellofemoral ligament
POL: Posterior oblique ligament
TERA: Tibial external rotation angle
TLR: Triangular-vector ligament reconstruction
